# Computational Modeling Meets 3D Bioprinting: Emerging Synergies in Cardiovascular Disease Modeling

**DOI:** 10.1002/adhm.202503034

**Published:** 2026-02-15

**Authors:** Tanmay Mukherjee, Mehdi Salar Amoli, Sarah Rezapourdamanab, Lama Rita El Shammas, Martin L. Tomov, Emilio A. Mendiola, Vahid Serpooshan, Reza Avazmohammadi

**Affiliations:** ^1^ Department of Biomedical Engineering Texas A&M University College Station TX USA; ^2^ Wallace H. Coulter Department of Biomedical Engineering Emory University School of Medicine and Georgia Institute of Technology Atlanta GA USA; ^3^ Children's Heart Institute McGovern Medical School UTHealth Houston TX USA; ^4^ Department of Cardiovascular Sciences Houston Methodist Academic Institute Houston TX USA; ^5^ J. Mike Walker ’66 Department of Mechanical Engineering Texas A&M University College Station TX USA

**Keywords:** bioprinting, cardiovascular diseases, computational fluid dynamics, in vitro models

## Abstract

Cardiovascular diseases (CVDs) remain the leading cause of death worldwide, underscoring the need for improved strategies in diagnosis, treatment, and disease modeling. Traditional in vitro models often fall short in replicating human CV physiology, prompting efforts to advance cardiac tissue engineering and computational modeling. Among these, three‐dimensional (3D) bioprinting has emerged as a transformative tool, enabling the creation of biomimetic CV constructs that more faithfully replicate native tissue structure and function. However, challenges persist in achieving appropriate mechanical properties and long‐term performance of engineered CV constructs. Computational modeling offers powerful solutions to assist with these challenges, providing predictive insights into structural remodeling, hemodynamics, disease progression, and therapeutic response. Techniques such as computational fluid dynamics and machine learning are increasingly used to optimize design and simulate physiological conditions. The integration of computational models with 3D bioprinting has led to hybrid platforms that enhance the precision and utility of engineered tissues. This review highlights recent advances in computational modeling applied to 3D bioprinted CV constructs, focusing on the added benefits of integrating these technologies to achieve a more accurate modeling of complex CV conditions. Together, these technologies offer a promising path toward clinically translatable, patient‐specific CV platforms.

## Graphical abstract

Emerging advances in three‐dimensional bioprinting and computational modeling are reshaping cardiovascular (CV) research by enabling more realistic, patient‐specific tissue platforms. This review surveys cutting‐edge approaches that merge biomimetic CV constructs with computational simulations to overcome the limitations of traditional models, improve physiological relevance of in vitro platforms, and accelerate the development of clinically translatable diagnostic and therapeutic strategies.

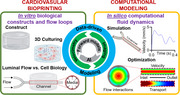

**Abbreviations**
0D: zero dimension
1D: one‐dimensional
2D: two‐dimensional
3D: three‐dimensional
ANN: artificial neural network
BC: boundary conditions
CAD: computer‐aided design
CAVD: calcific aortic valve disease
CFD: computational fluid dynamics
CHD: congenital heart disease
CM: cardiomyocytes
CNN: convolutional neural network
CT: computed tomography
CV: cardiovascular
CVD: cardiovascular disease
DCM: dilated cardiomyopathy
DLP: digital light processing
DNN: deep neural network
EC: endothelial cell
ECM: extracellular matrix
EndMT: endothelial‐to‐mesenchymal transition
FALDF: ontan‐associated liver disease
FE: finite element
FRESH: freeform reversible embedding of suspended hydrogels
FSI: fluid structure interactions
GelMA: gelatin methacrylate
hiPSC: human induced pluripotent stem cell
HLHS: hypoplastic left heart syndrome
ICASA: internal carotid artery sidewall aneurysm
LDV: laser Doppler velocimetry
LV: left ventricle
MAPCA: major aortopulmonary collateral arteries
MI: myocardial infarction
ML: machine learning
MRI: magnetic resonance imaging
NS: Navier–Stokes
ODE: ordinary differential equation
PAH: pulmonary arterial hypertension
PAS: pulmonary artery stenosis
PC: phase contrast
PDMS: polydimethylsiloxane
PEG: polyethylene glycol
PH: pulmonary hypertension
PINN: physics‐informed neural network
PIV: particle image velocimetry
RBF: radial basis function
SMC: smooth muscle cell
TAVI: transcatheter aortic valve implantation
TEBV: tissue‐engineered blood vessel
TEVG: tissue‐engineered vascular graft
VFI: vector flow imaging
VIC: valvular interstitial cell
WSS: wall shear stress

## 1 Introduction

Cardiovascular diseases (CVDs), a group of conditions affecting the heart and blood vessels, are the leading cause of death worldwide, accounting for 32% of global deaths in 2019 [1]. Some of the most significant types of CVDs include vessel narrowing, such as stenosis, atherosclerosis, coronary artery disease, hypoplastic left heart syndrome (HLHS), pulmonary hypertension (PH), and pulmonary vein stenosis [2, 3, 4, 5]. While primarily affecting the heart and blood vessels, CVDs can also initiate a cascade of complications in noncardiac organs, leading to conditions such as Fontan‐associated liver disease (FALD) [6] and cardiorenal syndrome [7, 8]. Treatments addressing CVD complications have drastically improved in recent decades; however, a range of challenges remain due to the inherent individual‐specific complexities of these diseases, as well as complexities rooted in the presence of comorbidities [9, 10]. Current diagnostic approaches primarily rely on anatomical assessments derived from imaging modalities, including echocardiography, computed tomography (CT), and magnetic resonance imaging (MRI). However, these techniques may fail to detect patients at high risk before the onset of clinical symptoms [11]. As a result, the first symptom of CVD in 50% of cases is an acute cardiac event or sudden cardiac death [12]. Many of these conditions eventually require surgical interventions, which are often quite complex, involving risks such as infection, bleeding, or stroke, and can result in lifelong complications. Achieving a full and clear understanding of the disease anatomy during these procedures is often difficult, which further complicates the development of an accurate and effective treatment plan [13, 14]. Moreover, the development of therapeutics for these clinical conditions often faces challenges, such as effective drug delivery or the emergence of resistance to certain therapeutics [15]. Treatment options, including both pharmaceutical and device therapies, also require fulfilling strict and rigorous regulatory steps [16, 17]. In response to these challenges, persistent effort has been directed toward preclinical research approaches at the interface of biology and engineering. These efforts aim to provide a deeper understanding of CVDs, refine diagnostic and prognostic techniques, and improve treatment options [18]. A variety of functional models have been developed to support these objectives, enabling deeper investigation into disease onset, progression, therapeutic interventions, and the performance of emerging drugs and medical devices [19, 20].

**FIGURE 1:**
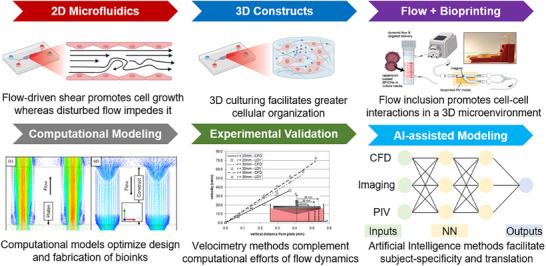
Overview of in vitro and in silico modeling techniques to facilitate investigations of cardiovascular physiology. The review details mathematical, biophysical, and statistical techniques used to simulate biological processes (e.g., cell growth and differentiation), inform biofabrication, and facilitate in vitro / ex vivo recapitulation of physiological and pathophysiological processes in vivo. CFD: computational fluid dynamics, PIV: particle image velocimetry, NN: neural network. Reproduced with permission [47]. Copyight 2024, John Wiley and Sons.  Reproduced with permission [48]. Copyright 2008, ASME. Reproduced with permission []. Copyright 2019, John Wiley and Sons.

The modeling techniques currently employed in CVD research encompass a range of approaches, including in vivo studies using various animal models [21], in vitro and ex vivo experimental strategies that recapitulate complex disease anatomy in laboratory settings [22, 23], and computational modeling [24]. In vitro and ex vivo modeling techniques have served as valuable complements to animal models to study CVDs (Figure 1). 2D culture models of CVD are extensively used, consisting of various combinations of CV (and noncardiac) cells, such as cardiomyocytes (CMs), fibroblasts, endothelial cells (ECs), smooth muscle cells (SMCs), and different stem cells. These 2D models enable relatively high‐throughput analysis of parameters such as cell viability and proliferation, cytokine release, gene expression profiles, electrophysiology, and rhythm disorders [25, 26]. However, such models are unable to replicate the intricate 3D cell–cell and cell–matrix interactions. Over the past decades, tissue engineering has provided promising alternatives to the conventional disease models informing drug/device development pipelines, where 2D cell cultures and/or animal models have traditionally been used to screen candidate treatments before clinical trials [27, 28, 29].

The field of tissue engineering has transformed rapidly in recent years, driven by the advent of technologies such as additive biomanufacturing and 3D bioprinting (Figure 1). Tissue bioprinting involves the use of a robotic system (printer) to deposit a mixture of cells, biomaterials, and support factors, known as bioink, layer‐by‐layer, to create the target 3D structure [28, 30, 31, 32]. Bioprinting has shown great promise in CV tissue engineering and regenerative medicine by creating functional, large‐scale 3D constructs, incorporating complex architectures, and heterogeneous cell populations [20, 33, 34]. Examples include 3D bioprinted blood vessel structures [35, 36], heart valves [37, 38], functional heart tissue (myocardium) analogs [39], and heart chambers [40] used to study normal development as well as various adult and congenital heart diseases (CHDs) [41, 42]. Numerous researchers have successfully used various bioprinting approaches to create engineered CV tissues that can maintain perfused vascular flow at physiological rates for extended culture periods in vitro, while maintaining the scaffold integrity and cardiac function [41, 43, 44, 45]. Tissue slices also offer a valuable ex vivo model for studying CVDs by preserving the native architecture, cell composition, and extracellular matrix (ECM) of the heart and vasculature [23, 46]. These thin, viable sections can be maintained in culture and subjected to mechanical, electrical, or pharmacological stimuli to replicate disease conditions. While these advancements in tissue engineering have greatly expanded the potential of in vitro and ex vivo CV models, integrating computational modeling offers a powerful complement, potentially further enhancing mechanistic insight, predictive power, and design optimization.

Computational modeling of CV diseases has advanced rapidly in recent years, significantly enhancing our ability to predict, diagnose, and treat a wide range of conditions [24]. Techniques such as computational fluid dynamics (CFD) [50, 51, 52] have been increasingly employed to understand and address CVD etiology, pathophysiology, and therapeutics [53, 54, 55]. CFD platforms offer a robust tool for simulating alterations in hemodynamics and mechanical stresses experienced by the CV system under various conditions, such as stenosis, HLHS, or pulmonary arterial hypertension (PAH) [54, 55, 56]. Due to the complex interplay of multiple cellular and environmental factors in CV pathophysiology, utilizing a single experimental model to replicate a particular CVD has often resulted in suboptimal predictive performance, which is inadequate for clinical applications [21, 57, 58]. Combining computational modeling with advanced tissue engineering and biomanufacturing techniques has therefore been explored to establish robust, novel modeling platforms that can more faithfully and accurately recapitulate the complex and dynamic interplay between cellular and microenvironmental factors involved in CVDs and CHDs (Figure 1) [59, 60, 61]. These hybrid platforms can facilitate the development of new and more effective treatments and therapies, thereby reducing reliance on existing animal models for preclinical testing. Furthermore, computational tools can be used to fine‐tune and optimize the design and manufacturing of bioengineered CV tissue models, hence improving their biomimicry, precision, and predictive capabilities. Together, these advances can revolutionize the diagnosis of CVDs, enhance drug discovery and medical device development, and facilitate the establishment of more effective surgical and nonsurgical interventions and therapies for these diseases.

## 2 Modeling CV Physiology and Hemodynamics with In Vitro Constructs

### 2.1 2D In Vitro Platforms to Study Mechanotransduction

A range of in vitro and ex vivo techniques has been developed to model CV hemodynamics and flow‐induced mechanotransduction. Various flow chamber devices have been designed and utilized as in vitro fluidic systems to deepen understanding of CV cell responses to varying flow conditions [62, 63]. The first group of such devices examines the impact of flow on monolayer cultures of cells. Cone‐and‐plate devices are among the most commonly used platforms for applying controlled shear stress stimulations to cell monolayers, such as ECs [64]. The parallel‐plate flow chamber is another common experimental setup in which two plates are placed parallel, providing an open channel to guide the fluid flow [62, 65]. These devices often induce shear stress on cells, including ECs, and evaluate their response. Another monolayer culture model involves culturing cells in conventional tissue culture vessels on an orbital shaker. The circular fluid motion in these models applies various multiaxial and uniaxial shear stress regimes to the cell layer [66, 67]. The recapitulation of the native microenvironment of CV tissues has been dramatically improved through 2D microfluidic devices. Such methods typically offer greater precision, throughput, and reproducibility of flow parameters and patterns [68]. For example, microfluidic devices incorporating a stretchable membrane based on polydimethylsiloxane (PDMS) have been utilized to investigate the impact of physiological strain on vascular aging [69]. By enabling precise control of shear stress, flow patterns, and mechanical strain, these advanced 2D platforms provide powerful tools for dissecting the biomechanical drivers of CVD progression, laying the groundwork for more predictive and physiologically relevant in vitro models.

### 2.2 3D In Vitro Platforms to Study Cell–ECM Interactions

While 2D in vitro fluidic systems have found robust applications in CVD modeling, a more accurate study of CV cell function, as well as cell–cell and cell–ECM interactions, particularly in response to flow alterations, requires a 3D culture environment [18, 34]. Tissue engineering and biomanufacturing techniques have been increasingly applied to create more physiologically faithful 3D models of CV systems, achieving unprecedented structural, cellular, and functional complexity and biomimicry [70, 71]. The bioengineered 3D in vitro / ex vivo models of CV flow could be classified into four categories: spheroid culture platforms, cellular hydrogel scaffolds, tissue slice systems, and tissue‐engineered blood vessel models (including vessel‐on‐a‐chip platforms). Spheroids provide a significant advance in mimicking native tissue structure compared to conventional 2D cell culture models and have therefore been used to examine CVDs, such as atherosclerosis [71]. For instance, foam cell spheroids have been utilized by multiple groups to evaluate the efficacy of various therapeutic compounds (e.g., sirolimus‐loaded microparticles) on foam cell formation and associated inflammation in the context of atherosclerotic plaque formation [72, 73]. While these models offer high throughput and generate large datasets of cellular processes, their application under dynamic flow conditions remains limited.

### 2.3 Integration of Fluid Flow Facilitates the Recapitulation of Biological Processes

Cellular hydrogel platforms offer a versatile and increasingly sophisticated approach for modeling CV tissue in a 3D environment that closely resembles native ECM conditions [74]. These platforms involve encapsulating CV cell types, such as ECs, smooth muscle cells, and cardiomyocytes, within natural or synthetic hydrogels that provide mechanical support and biochemical cues necessary for tissue‐specific function. Hydrogels derived from materials such as collagen, fibrin, gelatin methacrylate (GelMA), or polyethylene glycol (PEG) can be tailored in terms of stiffness, porosity, and degradation rate to simulate the dynamic biomechanical environment of the CV system [75, 76]. Importantly, these scaffolds allow for the integration of flow by embedding them within microfluidic or bioreactor systems, enabling researchers to study how hemodynamic forces influence cell behavior, matrix remodeling, and disease progression. Such platforms have been used to investigate various aspects of CV pathophysiology, including endothelial barrier function, neointimal hyperplasia, and cardiac fibrosis [77, 78]. By facilitating 3D spatial organization and more physiologically relevant cell–matrix and cell–cell interactions, cellular hydrogel models serve as a crucial bridge between simple 2D systems and complex tissue constructs, thereby advancing our understanding of CVD mechanisms and therapeutic responses.

Tissue slice systems provide an ex vivo platform that preserves native architecture, multicellular composition, and ECM organization of the myocardium, offering a unique biomimetic environment for studying CVDs. These thin, viable sections, prepared from human or animal heart or vessel tissue, allow for precise spatial and temporal investigation of cardiac or vascular function under controlled conditions [79, 80]. When maintained in specialized culture chambers or perfusion bioreactors, tissue slices can remain viable for extended periods and be subjected to electrical, mechanical, or pharmacological stimuli to replicate physiological or pathological conditions. This has enabled detailed studies of myocardial contractility, electrophysiological properties, cellular remodeling, and drug responses in the context of various disease states, including heart failure, myocardial infarction (MI), and cardiomyopathies [46, 81, 82]. Compared to isolated cell models or organoids, tissue slice systems offer greater anatomical fidelity, retaining the complex intercellular and matrix interactions critical to understanding disease progression and therapeutic outcomes. However, a key limitation of tissue slice systems is that, while they preserve native 2D architecture and cell–matrix interactions, they lack the full 3D structural and mechanical complexity of intact CV tissues, limiting their ability to fully recapitulate complex in vivo adaptations.

Tissue‐engineered blood vessel models, including vessel‐on‐a‐chip platforms, have emerged as powerful tools for studying vascular biology and pathophysiology under controlled and physiologically relevant conditions. These systems are typically fabricated using biocompatible scaffolds or hydrogels seeded with vascular cells, such as ECs and SMCs, and perfused with fluid to replicate hemodynamic forces present in vivo [83, 84]. Blood vessel models have been used to investigate a wide range of CV conditions, including atherosclerosis, aneurysms, and thrombosis, as well as to assess the efficacy and safety of pharmaceuticals [85, 86, 87]. Recent advances in microfluidics have enabled the development of vessel‐on‐a‐chip platforms that incorporate multiple vascular cell types, dynamic flow, and mechanical strain to mimic key aspects of vascular function and disease. These microphysiological systems enable precise control of microenvironmental parameters and facilitate the study of cellular responses to complex stimuli, including disturbed flow and inflammatory cytokines. Moreover, patient‐derived cells have been incorporated into these platforms to model genetic or acquired vascular diseases in a personalized context. Despite their versatility and growing impact, many tissue‐engineered blood vessels (TEBVs) and vessel‐on‐a‐chip models still face challenges in replicating the long‐term remodeling processes and the hierarchical architecture of native vascular networks.

## 3 3D Bioprinted Models to Study CV Development, Disease Processes, and Hemodynamic Pathologies

**FIGURE 2:**
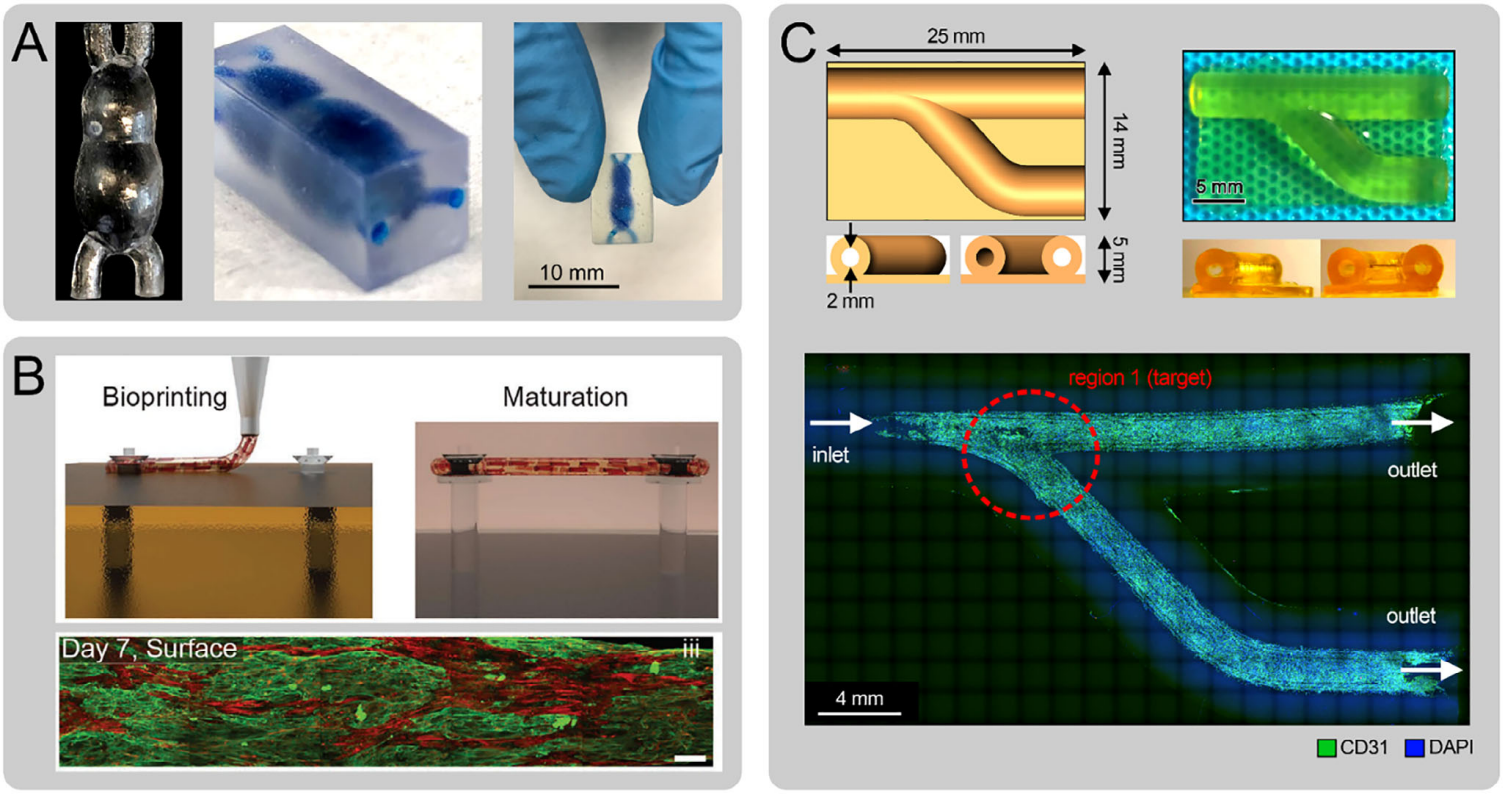
Examples of the various applications of 3D bioprinting in CVD modeling. (A) 3D printed model of developing heart tube, created based on a predesigned computer‐aided design (CAD) model. Reproduced with permission [41]. Copyright 2020, John Wiley and Sons (B) Fabrication of aligned cardiac fibers through 3D bioprinting. Reproduced with permission [88]. Copyright 2022, John Wily and Sons. Scale bar: 100 µm. (C) 3D bioprinted model of pulmonary vein stenosis to assess therapeutic delivery strategies using magnetic nanoparticles. Reproduced with permission [47]. Copyright 2024, John Wiley and Sons.

3D bioprinted models represent a transformative advancement in in vitro CV research, offering greater control over spatial organization and cellular complexity compared to traditional 2D cultures and conventional 3D constructs (Table 1). While earlier models have been instrumental in advancing our understanding of cell behavior and disease mechanisms, they often lack the architectural fidelity and tissue heterogeneity of native CV structures. Bioprinting enables the layer‐by‐layer deposition of multiple cell types and biomaterials with microscale precision, thereby creating complex, vascularized tissues that closely mimic in vivo anatomy and function. These engineered tissues can recapitulate key developmental and pathological processes, making them powerful platforms for studying CV development, disease progression, and therapeutic response. Examples of such applications include modeling human heart development (Figure 2A) [41], fabrication of aligned cardiac muscle fibers (Figure 2B) [88], and the study of pulmonary vein stenosis and therapy (Figure 2C) [47].

**TABLE 1:** The use of 3D bioprinted in vitro models to study CV development and disease processes.

Focus Area	Bioprinting Method	Cell(s)	Bioink(s)	Main Findings
Human heart early development [41]	Extrusion bioprinting with perfusion bioreactor	HUVECs	Gelatin methacryloyl (GelMA) hydrogel	Replicated flow dynamics; supported EC growth in embryonic/fetal heart models
Human heart early development [89]	FRESH bioprinting	hiPSC‐CMs and cardiac fibroblasts	Collagen type I hydrogel	Synchronized contractions and functional assessment of embryonic heart tube
Hypoplastic left heart syndrome & normal fetal heart development [90]	3D printing using stereolithography based on fetal echocardiogram data	None	Photopolymer resin (FormLabs Clear Resin)	Studied flow differences between normal and HLHS fetal hearts
Human embryonic heart tube development [91]	DLP‐based 3D bioprinting	hiPSC‐CMs, HUVECs	Photocross‐linkable hydrogel mimicking cardiac ECM	Perfusable heart tube with viability and contractility
Tetralogy of Fallot with MAPCAs [43]	SLA‐based 3D bioprinting	None	GelMA	Patient‐specific MAPCAs models used to simulate PAS and testing recanalization
Modeling LV myocardial fiber orientation [92]	Extrusion bioprinting with dECM hydrogel	hiPSC‐CMs and cardiac fibroblasts	dECM hydrogel	Replicated myocardial fiber orientation and synchronized contraction
Modeling of myocardial fiber orientation [93]	Focused rotary jet spinning	Neonatal rat ventricular cardiomyocytes	Polycaprolactone (PCL) fibers	Studied fiber misorientation effects on cardiac function
Myocardial infarction in aged heart [94]	Extrusion‐based 3D bioprinting using a tri‐printhead system	Aged hiPSC‐CM, EC, and CMF	GelMA, MeHA, aged type I collagen, and photoinitiator	Modeled aged myocardium post‐MI for regenerative therapy testing
Cardiac arrhythmia modeling [95]	Extrusion bioprinting of optoelectronically active scaffolds	hiPSC‐CMs	GelMA embedded with silicon micro‐solar cells	Enabled wireless light‐modulated cardiac beating
Arrhythmia, myocardial infarction [96]	Aspiration‐based bioprinting	hiPSC‐derived CMs, fibroblasts	Self‐healing Hydrogel	fabricated high‐density cardiac microtissues exhibiting features of scarred myocardium post‐MI
Pulmonary artery stenosis associated with Williams syndrome [97]	Extrusion‐based 3D bioprinting	hiPSC‐ECs derived from Williams syndrome patients	GelMA	Studied EndMT under altered flow to better understand PAS
Pulmonary artery atresia [44]	SLA‐based 3D bioprinting	Human endothelial cells	GelMA	Modeled vascular anastomosis and identified stenosis‐prone areas
Pulmonary vein stenosis [47]	Extrusion bioprinting with perfusion bioreactor	HUVECs	GelMA with rapamycin‐loaded nanoparticles	Used nanoparticles to reduce EC proliferation in PVS
Atherosclerosis [98]	Coaxial 3D bioprinting	HUVECs	Vascular tissue‐specific	Mimicked early atherosclerosis with inflammatory features
Atherosclerosis [99]	3D bioprinting of patient CT scans	None	None	High‐resolution analysis of fluid dynamics to study vascular diseases
Coronary artery disease [100]	3D bioprinting	ECs	Poly(d,l‐lactide)	Supported endothelial cell growth and reduced restenosis risk
Aortic valve disease [101]	Silicone additive manufacturing	None	UV‐cured Silicone	Showed hemodynamic performance comparable to TAVI devices and healthy valves
Fontan‐associated liver disease [102]	3D bioprinting (chip model)	Hepatocytes, Stellate Cells, ECs	Multilayered bioinks	Replicated FALD flow conditions with fibrosis features
Hypertensive nephropathy [103]	Microfluidic organ‐on‐a‐chip platform with dual‐channel design	Human glomerular endothelial cells and podocytes	ECM‐coated porous membrane	Increased mechanical forces lead to cytoskeletal rearrangement and damage to cell junctions
Large vessel replacement [104]	3D bioprinting on rotating mandrel	Rat fibroblasts and SMCs	Scaffold‐free approach using only cells	Implanted rat aortas integrated and functioned like native vessels
Regenerative medicine & drug screening [105]	Hybrid extrusion‐ bioprinting with microfluidic perfusion	hiPSC‐CMs and HUVECs	Composite bioinks, microfibrous hydrogel	Created endothelialized, aligned, contractile myocardium
Cardiac tissue engineering; modeling myocardial anisotropy [88]	Extrusion bioprinting using anisotropic organ building blocks	hiPSC‐CMs	Fibrin‐based hydrogel	Constructs with anisotropic contractile force & conduction velocity

A variety of 3D bioprinting technologies and strategies have emerged and been applied for the fabrication of CV tissue analogs. Among these, extrusion‐based bioprinting remains the most widely adopted modality. In this approach, shear‐thinning hydrogel filaments laden with cells and biomolecules are deposited in a layer‐by‐layer fashion, enabling the fabrication of volumetric constructs with spatial control [106, 107, 108]. The versatility of extrusion bioprinting permits the design of constructs that feature gradients of biomaterials, biomolecular species, cellular populations, and mechanical properties such as stiffness, all of which can significantly influence cellular behavior by modulating cell‐cell and cell‐matrix interactions and affecting tissue maturation [109, 110]. A broad range of biomaterials, including gelatin, alginate, chitosan, hyaluronic acid, collagen, silk, cellulose derivatives, as well as synthetic polymers such as PEG and Pluronic, can be formulated into printable bioinks for extrusion bioprinting, making these strategies highly adaptable to various CV tissue engineering applications [111, 112]. Critical enhancements to basic extrusion modalities have expanded their suitability for bioprinting of CV‐relevant architectures. For example, the freeform reversible embedding of suspended hydrogels (FRESH) or the embedded printing method enables deposition of cell‐laden hydrogel filaments into a support bath, which maintains filament geometry until cross‐linking is complete. This enables the fabrication of overhanging, unsupported hollow or branching structures, such as vascular networks, using relatively simple hardware. A growing body of research reports the use of FRESH to generate cardiac chamber‐like structures and hierarchical vascular networks for in vitro modeling of CVD and drug testing, printing vessel diameters from ∼1 mm down to ∼250 µm [113, 114]. This approach has enabled bioprinting of perfusable constructs, improved nutrient/oxygen transport, and deeper recapitulation of microenvironmental cues. Another enhancement, coaxial extrusion printing, utilizes concentric nozzles to fabricate filaments with distinct core–shell compositions (e.g., an inner lumen‐forming sacrificial ink or an endothelial‐cell‐laden shell) that can be deployed to generate vascular conduits or endothelial‐lined tubes in a single step [115, 116]. These strategies enhance the relevance of bioprinted constructs for CVD modeling, including modeling of vascular pathologies, perfusion studies, and drug screening.

Despite their widespread use, extrusion‐based bioprinting techniques have inherent limitations when used for CV modeling. These include comparatively low spatial resolution (typically filament diameters on the order of hundreds of micrometers), slower printing speeds, mechanical shear stresses imposed on cells during extrusion (which may impair viability or phenotype), and challenges in achieving highly complex architectures without substantial support scaffolds or sacrificial templates [117, 118, 119]. To overcome some of these constraints, light‐based bioprinting strategies have gained considerable attention. Light‐based strategies, such as digital light processing (DLP) and, to a lesser extent, stereolithography (SLA), employ a projected light source (UV or visible light) to polymerize a photocurable bioink layer by layer. Because each layer is cured as a whole, these methods offer significantly higher spatial fidelity and shorter build times than extrusion. In cardiac tissue engineering, this higher resolution enables the fabrication of more geometrically sophisticated structures, including microscale features that better mimic native myocardial anisotropy, microvasculature, or alignment cues. Still, repeated exposure to UV (or high‐intensity visible) irradiation within these strategies, combined with the presence of photoinitiators, can potentially induce cellular stress responses, DNA damage, or alter gene expression profiles. Furthermore, the high degree of cross‐linking often achieved in these systems may result in stiffer scaffolds that are less favorable for cell remodeling, maturation, or long‐term functional integration [120, 121, 122]. In the field of CV bioprinting, DLP bioprinting has been used with various cell sources, such as human‐induced pluripotent stem cell‐derived cardiomyocytes (hiPSC‐CMs), to develop cardiac tissue replicas in a high‐throughput format, demonstrating feasibility for treatment testing [114, 123]. Importantly, for disease modeling or drug screening applications, the faster throughput and higher resolution of DLP can be advantageous because smaller feature sizes and patterned alignment more closely simulate the physiological microenvironmental cues, enabling improved functional maturation and contractile behavior of printed constructs [124].

### 3.1 Bioprinted Models of Heart Development and Homeostasis

By precisely depositing bioinks composed of cells, biomaterials, and signaling molecules into defined geometries, researchers can recreate key features of early heart development, including chamber formation, cell fate specification, and tissue patterning (Table 1). Using medical imaging data, Cetnar et al. developed patient‐specific 3D bioprinted models of the developing human heart [41]. This study demonstrated the first anatomically accurate, 3D bioprinted models of the human heart at early developmental stages, specifically the embryonic heart tube (day 22) and the fetal left ventricle (LV) (week 33), using 3D bioprinting and perfusion bioreactor technologies. These models replicated physiologically relevant flow dynamics and supported EC growth, demonstrating the influence of geometry and flow on cellular behavior. Such approaches could elucidate the effects of hemodynamic alterations on normal human heart development and on various abnormal processes, i.e., the onset of CHDs. Similarly, Bliley et al., 3D bioprinted a simplified, bioinspired model of the human embryonic heart tube using the FRESH technique [89]. The bioprinted constructs, populated with human embryonic stem cell‐derived CMs and cardiac fibroblasts, exhibited synchronized contractions within days, maintained functionality for up to a month, and supported perfusion. Functional assessment revealed organized CM networks, anisotropic calcium transient, and measurable fluid displacement, establishing this model as a foundational step toward engineering more complex developing heart models. These studies underscore how 3D bioprinting can be used to reconstruct the spatial and temporal complexity of cardiogenesis in a controllable, high‐throughput laboratory setting.

In the context of CV homeostasis, 3D bioprinted heart tissues provide dynamic and physiologically relevant models to study how cells maintain structural and functional balance over time. A recent study by Zhang et al. utilized a hybrid 3D bioprinting approach to create endothelialized myocardium using composite bioinks and microfibrous hydrogel scaffolds, resulting in contractile, aligned cardiac tissues [105]. Integrated into a perfusion bioreactor, the system supported hiPSC‐derived CMs. A novel approach presented by Ahrens et al. bioprinted anisotropic organ building blocks composed of hiPSC‐CMs to fabricate aligned cardiac tissues with complex geometries [88]. The resulting constructs exhibited anisotropic contractile force and conduction velocity, demonstrating precise spatial control over tissue alignment and function, key indicators of tissue homeostasis. Ershad et al. presented an optoelectronically active scaffold made of GelMA embedded with micro‐solar cells and seeded with CMs to enable light‐stimulable, noninvasive modulation of cardiac beating [95]. The scaffold successfully maintained high cell viability and accelerated heartbeats without the need for wires or genetic modifications, offering a promising approach for wireless control of electrically active tissues. The study by Hwang et al. showed how bioprinting could be used to create complex, multioriented engineered heart tissues with synchronized contractions [92]. Their novel method successfully replicates the native cardiac architecture, including the myocardial fiber orientation within the LV. Interestingly, Chang et al. developed a focused rotary jet spinning technique to create polymeric scaffolds with controlled fiber orientation, enabling the fabrication of both healthy and diseased heart models [93]. Fabricated models, seeded with contractile cells, allowed for the study of how fiber misorientation affects cardiac function.

Several groups have also examined bioprinting functional coronary vascular structures. For instance, Dell et al. demonstrated the creation of bioprinted rat aortas using fibroblasts and SMCs on a rotating mandrel [104]. These 3D bioprinted vessels were successfully implanted into rats, where they integrated with native vasculature and native vessel‐like function. Their findings underscore the potential of bioprinted vascular grafts for future human vascular repair. In sum, bioprinted in vitro models of CV systems are increasingly developed and employed, particularly, to probe how perturbations, genetic or biomechanical, can disrupt homeostasis and potentially initiate disease processes.

### 3.2 Bioprinted Models of CHDs

CHDs are among the most common birth defects, affecting millions globally and significantly impacting morbidity and mortality rates. Nonbiological 3D printing has been well established in the CHD field, enhancing surgical planning, disease visualization, and education by providing customizable, accurate models that improve procedural precision, help patients understand their conditions, and offer valuable resources for medical training [125, 126]. More recently, 3D bioprinted models have emerged as powerful tools in studying various CHDs, providing a platform for more accurate in vitro representation of pathophysiological processes (Table 1) [45, 127]. These congenital anomalies manifest with various structural and functional impairments affecting the heart's chambers, valves, and vasculature. Bioprinted models can allow for the investigation of how disruptions in the cellular architecture or ECM / microenvironmental components contribute to CHD pathogenesis [45, 110].

HLHS, a condition in which the left side of the heart, including the LV, mitral valve, aortic valve, and ascending aorta, is severely underdeveloped, is one of the most critical and life‐threatening CHDs, often leading to complex clinical challenges and requiring multiple staged surgical interventions [128, 129]. To better understand and potentially treat HLHS, researchers have created patient‐specific 3D printed heart models using imaging data of the developing heart. Falk et al. developed a patient‐specific 3D printed fetal heart model from echocardiogram data, and integrated 4D flow MRI to study flow differences between normal and HLHS fetal hearts [90]. While their models could not support dynamic cell culture and analysis, newer techniques such as stereolithography and embedded bioprinting offer improved biocompatibility and fidelity, enabling more functional soft tissue models. For instance, Jin et al. created a perfusable model of human embryonic heart tube using DLP‐based 3D bioprinting and hiPSC technology [130]. This model replicated key structural layers of the early heart and was populated with human iPSC‐CMs and ECs, exhibiting tissue viability and contractility. The bioprinted heart tube is suggested as a powerful platform to study the effects of microenvironmental factors (e.g., flow perturbations) on the pathogenesis of various CHDs, including HLHS. Such models hold the potential to help differentiate the impact of altered hemodynamics from genetic programming, paving the way for novel therapies and interventions targeting HLHS‐specific development.

Studying (re)stenosis in the pulmonary vasculature is particularly challenging in CHDs due to its highly patient‐specific nature, stemming from factors like surgical alterations, shunt‐related flow changes, PH‐induced remodeling, or idiopathic causes [131, 132, 133, 134]. 3D bioprinting technologies allow the modeling of complex pulmonary artery stenosis (PAS) and pulmonary vein stenosis (PVS) using patient‐specific geometries and cellularized bioinks. These models, particularly those with bilayer vascular structures, enable investigation of EC and SMC signaling, especially processes such as endothelial‐to‐mesenchymal transition (EndMT) implicated in vascular remodeling and stenosis. 3D bioprinted models, together with perfusion bioreactor systems, enable exploration of how specific anatomical variations and hemodynamic patterns contribute to cellular changes in the context of these congenital vascular diseases [47, 125]. PAS is commonly linked to genetic mutations, but long‐term treatment remains ineffective due to its varied causes [135, 136]. Researchers have identified hemodynamic forces as a significant contributor to stenosis via EndMT. To better understand PAS, Saadeh et al. recently reported the use of a 3D bioprinted model that simulated patient‐specific conditions, utilizing hiPSC‐derived cells from Williams syndrome patients to study how altered flow regimens promote EndMT [97]. In diseases such as PH, where vascular cell layers become hyperplastic, 3D culture systems have proven valuable for dissecting EndMT‐driven mechanisms of smooth muscle overgrowth and vessel occlusion [137, 138].

Vascular atresia is typically treated by transcatheter recanalization or surgical anastomosis, but the cellular responses to these procedures remain poorly understood. In a recent study, Tomov et al. introduced a novel in vitro platform to model pulmonary artery anastomosis, using 3D bioprinted models of arteries, seeded with ECs to simulate vascular reconnection. Flow velocity and shear stress were quantified to identify areas prone to stenosis. The study highlighted how vascular geometry and flow influence EC function and could enhance surgical planning for diseases caused by disturbed flow [44].

Bioprinted models of CHDs could also be employed to develop and/or optimize various drug delivery and targeting processes, prior to translating the novel platforms into preclinical and clinical studies. For instance, Ning et al. recently reported the use of a 3D bioprinted in vitro model of PVS, consisting of bifurcated channels seeded with ECs, to examine targeted delivery of nanotherapeutics. By incorporating functional, rapamycin‐loaded nanoparticles, perfusion bioreactors, and an external magnetic field, the in vitro bioprinted model demonstrated the ability to target and reduce EC proliferation at high‐risk bifurcation sites, while minimizing the off‐target toxicity [47]. This approach showed promise for studying PVS (and other vascular anomalies) and developing pharmacotherapies, offering a more patient‐specific and tunable platform for safe and efficient clinical translation of drug screening efforts.

3D printing and bioprinting offer significant advantages in addressing CHDs by enabling the creation of patient‐specific vascular models for interventional planning and optimization. These technologies enable detailed simulation of complex heart anatomy, improving procedural accuracy and reducing risk. By visualizing anatomical variations and practicing interventions on bioprinted models, clinicians can enhance their understanding, refine techniques, and develop novel strategies for treating vascular anomalies. This approach promises to improve patient outcomes and facilitate the development of personalized, targeted therapies. For instance, Tomov et al. 3D printed and bioprinted patient‐specific in vitro models of tetralogy of Fallot with major aortopulmonary collateral arteries (MAPCAs). These models were used to simulate PAS, test recanalization techniques, and analyze flow dynamics with contrast agents and CFD [43]. This approach provides a valuable tool for training and developing new interventional therapies for vascular anomalies.

3D bioprinting has also been used to create vascular grafts tailored for pediatric CHD repairs. While traditional tissue‐engineered vascular grafts (TEVGs) have limitations, particularly in matching the unique anatomical and growth needs of children, 3D bioprinting offers a promising solution by creating highly precise, patient‐specific grafts with complex geometries tailored to individual anatomical needs, which is particularly important for pediatric patients whose CV structures are unique and constantly growing [104, 139]. Unlike conventional grafts, which may suffer from thrombosis, immunogenicity, limited growth potential, and poor integration, 3D bioprinted grafts can overcome these limitations by incorporating live (patient‐derived) cells, bioactive materials, and enhanced mechanical properties, offering a more adaptable and long‐lasting solution [140, 141].

CHDs are frequently complicated by complex valvular abnormalities, which differ from adult valvular disease in etiology and presentation. While adult cases often involve degeneration, CHD‐related valvular issues stem from structural malformations leading to stenosis or regurgitation [142, 143]. 3D printing and bioprinting offer powerful tools for visualizing valve anatomy, improving procedural planning, and predicting complications, especially in pediatric patients, where anatomical distortions and growth potential are key concerns [144, 145]. Efforts to bioprint implantable valves with the potential for somatic growth show promise, aiming to provide longer‐lasting and patient‐specific solutions for congenital valve disorders.

Patient‐specific 3D bioprinted models derived from imaging data and stem cells offer the possibility of testing individualized treatment strategies before clinical implementation. For example, drug efficacy or surgical interventions can be tested on 3D bioprinted models created from a patient's own cells, ensuring more targeted and effective treatments for CHDs [45, 146, 147]. The continued advancement of 3D bioprinting technology has the potential to revolutionize the study, diagnosis, and treatment of CHDs, leading to improved outcomes for affected patients.

### 3.3 Bioprinted Models of Acquired CVDs

3D bioprinted in vitro platforms could be employed to model various types of CVDs, including coronary artery and vascular disease, arrhythmia, structural heart defects, and acute conditions such as MI (Table 1). Coronary artery disease, along with cerebrovascular and peripheral arterial diseases, involves the narrowing or blockage of blood vessels due to plaque buildup (atherosclerosis), leading to impaired oxygen delivery to tissues. Treatment options for coronary artery diseases commonly include revascularization techniques such as angioplasty and stenting, alongside medications like antiplatelet agents and statins. 3D printing and bioprinting are increasingly contributing to these interventions, particularly in the development of next‐generation stents. For instance, Lu et al. created a 3D‐printed bioresorbable stent for the treatment of cerebrovascular conditions [100]. This stent supported EC growth, reduced restenosis risk, and safely degraded after vessel healing, highlighting the promise of bioprinted devices in vascular repair.

In a series of seminal works, the Cho group showcased the advancement of coaxial 3D bioprinting techniques to create freestanding, perfusable, and functional in vitro vascular models. The group first demonstrated the development of endothelialized vessels capable of mimicking key vascular functions and inflammatory responses, useful for disease modeling and tissue engineering [148]. In subsequent work, they developed a bioprinted arterial model using coaxial cell printing to create stable, three‐layered vascular tissues that mimic stenotic and tortuous arterial features. The model replicated early atherosclerotic events, such as endothelial dysfunction and inflammation, under turbulent flow conditions. It demonstrated that local flow dynamics regulate atherosclerosis initiation and allowed investigation of atorvastatin's dose‐dependent therapeutic effects, highlighting the model's potential for atherosclerosis research and drug screening. In another study, Maringanti et al. developed a 3D perfused microfluidic model (not bioprinted) of early‐stage human atherosclerosis using a coculture of SMCs, ECs, and foam cells within a fibrin‐collagen matrix [149]. The model maintained structural integrity under flow and exhibited hallmark features such as foam cell accumulation and immune cell recruitment. Circulating monocytes extravasated into the vessel wall, mimicking in vivo behavior. Gene expression confirmed disease‐specific markers, making this a valuable platform for studying atherosclerosis progression and therapeutic interventions. Antonowicz et al. used 3D printed artery models from patient CT scans to visualize blood flow in atherosclerosis using particle image velocimetry (PIV) [99]. The method enabled high‐resolution analysis of fluid dynamics, demonstrating its value for studying vascular disease. Together, these works demonstrate the potential of bioprinted vascular systems in studying vascular pathophysiology and drug development.

Rhythmic cardiac disorders have mostly been investigated using 2D and animal models. However, 3D bioengineering techniques, such as bioprinting, can create more realistic analogs of myocardial tissue, with sufficient contractile function, offering an alternative and robust platform to study arrhythmic events. A seminal work in this area, by Daly et al., employed an aspiration‐based bioprinting technique to create high‐cell density cardiac microtissues, replicating scarred tissue post‐MI with irregular electrical activity and reduced contractility [96]. The model was used to investigate arrhythmic behaviors, showing how altered cell ratios in the tissue led to disrupted electrical activity. This approach offers a platform for studying arrhythmias and testing treatments for electrical dysfunction in cardiac tissues. Another study, by Williams et al., developed a 3D human model of acquired cardiac arrhythmia using hiPSC‐CMs and cardiac fibroblasts [150]. Disrupting cell–cell signaling with methyl‐beta cyclodextrin‐induced arrhythmias, causing reduced conduction velocity, increased beating frequency, and fibrosis. Although 3D bioprinting was not employed in this study, it highlighted the potential of using 3D bioengineered models with patient‐specific cells, including those with genetic risks for arrhythmia, in drug development for arrhythmias.

Cardiomyopathies, a form of structural heart disease, impact the heart muscle by causing thickening of the heart walls (hypertrophic) or enlargement of the heart chambers (dilated). These conditions can be acquired through other health issues like arrhythmia or high blood pressure, but they can also be genetic. Treatment typically involves medication and lifestyle modifications, but in severe cases, heart transplants, ablation procedures, or surgical implants may be necessary. 3D bioprinting could offer a potential alternative for managing some of these severe cases. In particular, 3D bioprinted cardiac patch devices are increasingly investigated as an alternative regenerative medicine approach to treat ischemic cardiomyopathies (e.g., caused by MI) [151, 152]. Bioprinted constructs could also serve as in vitro modeling platforms to study the pathophysiology and treatment of various cardiomyopathies. Dilated cardiomyopathy (DCM), a leading cause of heart failure, involves heart enlargement and remains poorly understood due to limitations in current in vitro models. Traditional 2D systems fail to replicate the 3D structure and ECM changes seen in DCM. 3D bioengineered models, specifically the bioprinted ones, could better mimic the chemical, structural, and mechanical properties of diseased cardiac tissue [153]. These models enhance CM alignment, maturation, and function, offering promising tools to study DCM and develop new therapies. In addition to biological printing, synthetic 3D printing is increasingly used to visualize complex cardiac structures, aiding in the precise planning of treatments for various cardiomyopathies. For example, 3D printing has been used to visualize complex cardiac structures and to aid treatment planning in the management of hypertrophic obstructive cardiomyopathy. In one case, the patient's CT data was used to create 3D printed models, accurately reproducing the cardiac anatomy and assisting in the feasibility assessment of percutaneous transluminal septal myocardial ablation.

Cardiac valve disease, a structural heart defect, occurs when heart valves malfunction, leading to conditions like regurgitation or stenosis. Current models, such as animal studies and 2D cell cultures, have limitations in replicating human valve pathology and in simulating the complex 3D architecture and mechanical forces of native valve tissue. Recent developments in bioprinted cardiac valves aim to improve customization, biocompatibility, and functional performance by exploring advanced materials (bioinks) and cell‐based approaches. These innovations focus on creating personalized valve models that enhance tissue regeneration and replicate the mechanical properties necessary for effective clinical use. While many of these efforts concentrate on fabricating valve devices for replacement in vivo, several studies have utilized bioprinted valvular constructs for in vitro modeling [154]. For instance, van der Valk et al. developed a 3D bioprinted model of calcific aortic valve disease (CAVD) that mimicked the mechanical properties of human aortic valve leaflets using hydrogels and human valvular interstitial cells (VICs) [155]. The model was exposed to osteogenic media to induce microcalcification, enabling the study of VIC pathogenesis. This study provided insights into valvular mechanobiology and established a platform for high‐throughput drug screening for CAVD. Zeugin et al. also developed a bioinspired silicone aortic valve through precision 3D printing [101]. Compared with two transcatheter aortic valve implantation (TAVI) devices and a stenosed valve, the printed valve produced blood flow patterns similar to those of healthy aortic flow, thereby significantly reducing turbulent kinetic energy and energy loss.

3D bioprinted models can also enable faithful in vitro simulation of acute cardiac conditions, such as MI, by replicating cardiac structure, cell composition, and function. In the study by Daly et al., a bioprinting platform was introduced to create high‐density, spatially organized cardiac microtissues using self‐healing hydrogels, enabling disease modeling with high precision [96]. Using custom micropipettes, the spheroids were vacuum‐aspirated and transferred into the hydrogel. By controlling the CM‐to‐fibroblast ratio in spheroids, the team replicated fibrotic heart tissue after MI, capturing both impaired contractility and irregular electrophysiological behavior. The model allowed for the testing of microRNA therapies, particularly miR302b/c, which enhanced CM proliferation and improved both contraction and electrical integration, without promoting fibroblast growth. Importantly, the system supported early CM maturation, as shown by robust sarcomere formation and connexin‐43 expression, and offered a promising alternative to animal models for studying heart disease and screening regenerative therapies. Another study, by Basara et al., presented a 3D bioprinted model of aged human myocardium following MI, replicating the remote, border, and scar zones [94]. Using aged human cardiac cells and region‐specific bioinks, the model successfully mimicked the structural and functional properties of aged, fibrotic heart tissue, and demonstrated potential for evaluating regenerative therapies, such as stem cell‐derived extracellular vesicles.

### 3.4 Modeling Systemic Complications of CVD/CHD Using Bioprinted Constructs

3D bioprinted models could also be useful for studying CVD/CHD‐related complications in organs such as the kidneys, liver, and lungs, providing insights into systemic effects and disease progression. For example, liver models are used to study heart failure‐induced liver damage, particularly focusing on fibrosis and cirrhosis. One notable example is the study of FALD, in which bioprinted liver models enable researchers to examine how impaired cardiac function leads to liver congestion, inflammation, and fibrosis. These models help replicate the microenvironment of liver cells under the stress of reduced cardiac output, offering valuable insights into FALD pathophysiology. Rezapourdamanab et al. developed a 3D bioengineered FALD‐on‐a‐chip platform that mimicked the hepatic sinusoid structure [102]. They created 3D constructs using multilayered bioinks composed of hepatocytes, stellate cells, and ECs, and exposed them to flow conditions replicating healthy versus FALD‐like (elevated) pressures. Under disease‐mimicking flow conditions, the constructs exhibited altered cell morphology and reduced hepatic function, highlighting the model's ability to replicate key features of FALD. Such systems offer a valuable tool for studying hepatic fibrosis and testing potential treatments in a physiologically relevant environment. Similarly, 3D bioprinted kidney models can be employed to investigate how conditions, such as hypertension and hypertensive nephropathy, often associated with CVD, affect renal function and contribute to chronic kidney disease [103, 156, 157]. For instance, scalable 3D bioprinted models of kidney fibrosis have been developed, closely mimicking key cellular interactions driving chronic kidney disease [157]. Using human kidney cell lines, including epithelial, endothelial, and pericyte cells, these models can recreate the tubulo–interstitial architecture and support the development of fibrosis through epithelial injury and pericyte‐to‐myofibroblast differentiation, offering a promising platform for testing antifibrotic drugs. Further, Zhou et al. utilized a glomerulus‐on‐a‐chip model to recapitulate the human glomerular environment and study hypertensive nephropathy, revealing how mechanical forces cause cell damage and leakage [103]. Bioprinted lung models have been utilized to explore pulmonary complications in CVD patients, such as PAH and edema [137, 158]. These 3D models provide a more accurate understanding of disease mechanisms and enable the development of therapies targeting multiple organs affected by CV conditions. For instance, a 3D bioprinted model of pulmonary arterial adventitia was developed using a phototunable PEG‐based hydrogel and human pulmonary artery fibroblasts to mimic the mechanical and cellular features of PAH [137]. By controlling matrix stiffness, the model replicated fibrotic progression and cell proliferation seen in PAH, offering a promising platform to study disease mechanisms and screen therapies in vitro.

## 4 Computational Approaches to Recapitulating Physiological Conditions in In Vitro Settings

### 4.1 Parameterization of Design and Geometry for Bioprinting

**FIGURE 3:**
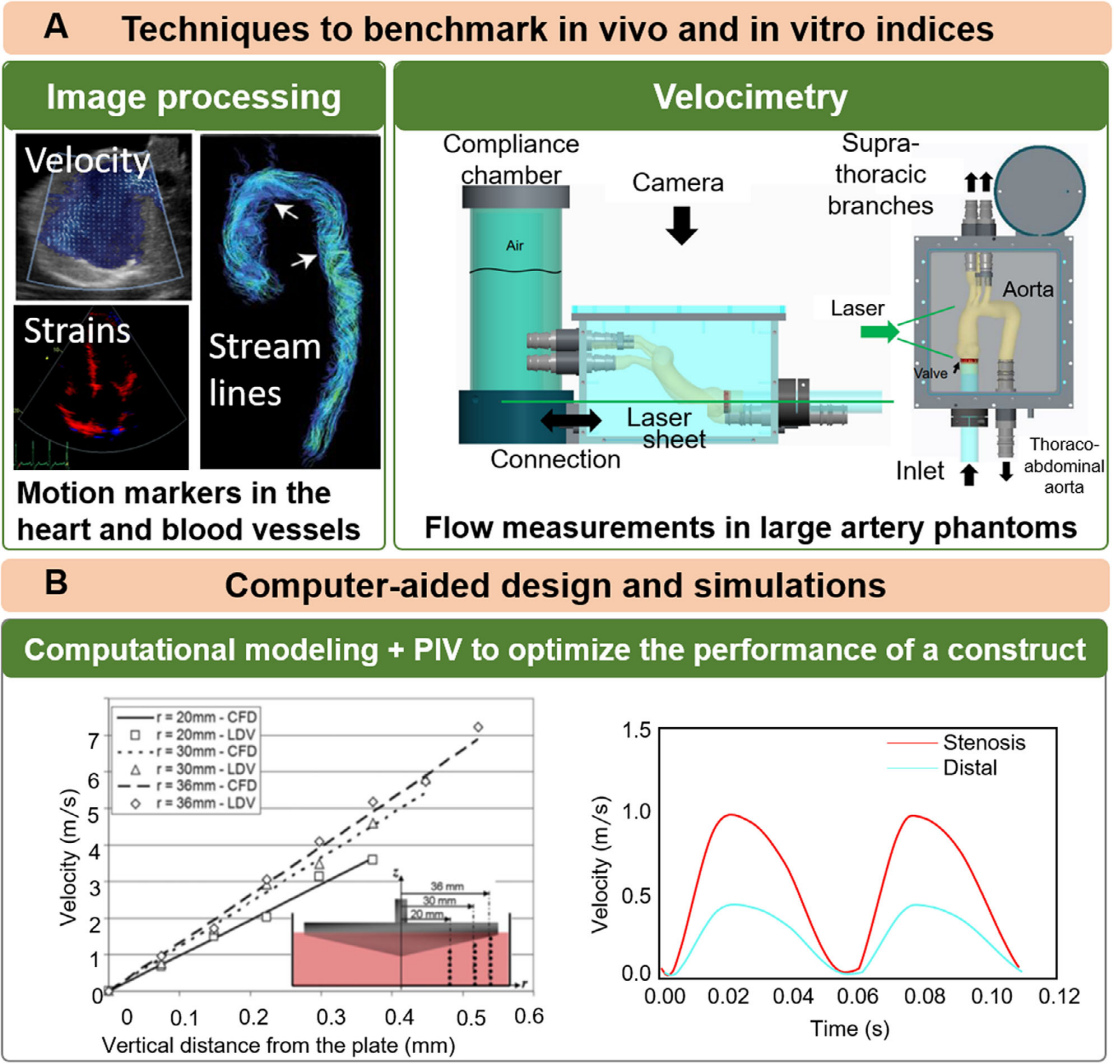
Integration of experimental and computational techniques to benchmark flow parameters for the optimal design and fabrication of biological constructs. (A) Measurements of intracardiac and vascular hemodynamics through motion estimation methods (left) in vivo, such as Doppler imaging, speckle tracking, and 4D Flow MRI, and (right) in vitro, using laser doppler velocimetry (LDV). (B) Velocimetry characteristics are used to inform computational models to extract additional biomarkers that are not readily accessible through experimental measurement to optimize different facets of construct performance. (Left) matching LDV velocities to optimize shear forces in an ex vivo culture and (right) recapitulate velocity profiles in anatomically‐relevant vascular settings. PIV: particle image velocimetry. Reproduced with permission [172], Copyright 2019, John Wiley and Sons.  Reproduced with permission [173], Copyright 2017, Springer Nature. Reproduced with permission [174], Copyright 2019, IEEE. Reproduced with permission [48]. Copyright 2008, ASME. and Reproduced with permission [125]. Copyright 2024, Frontiers.

**FIGURE 4:**
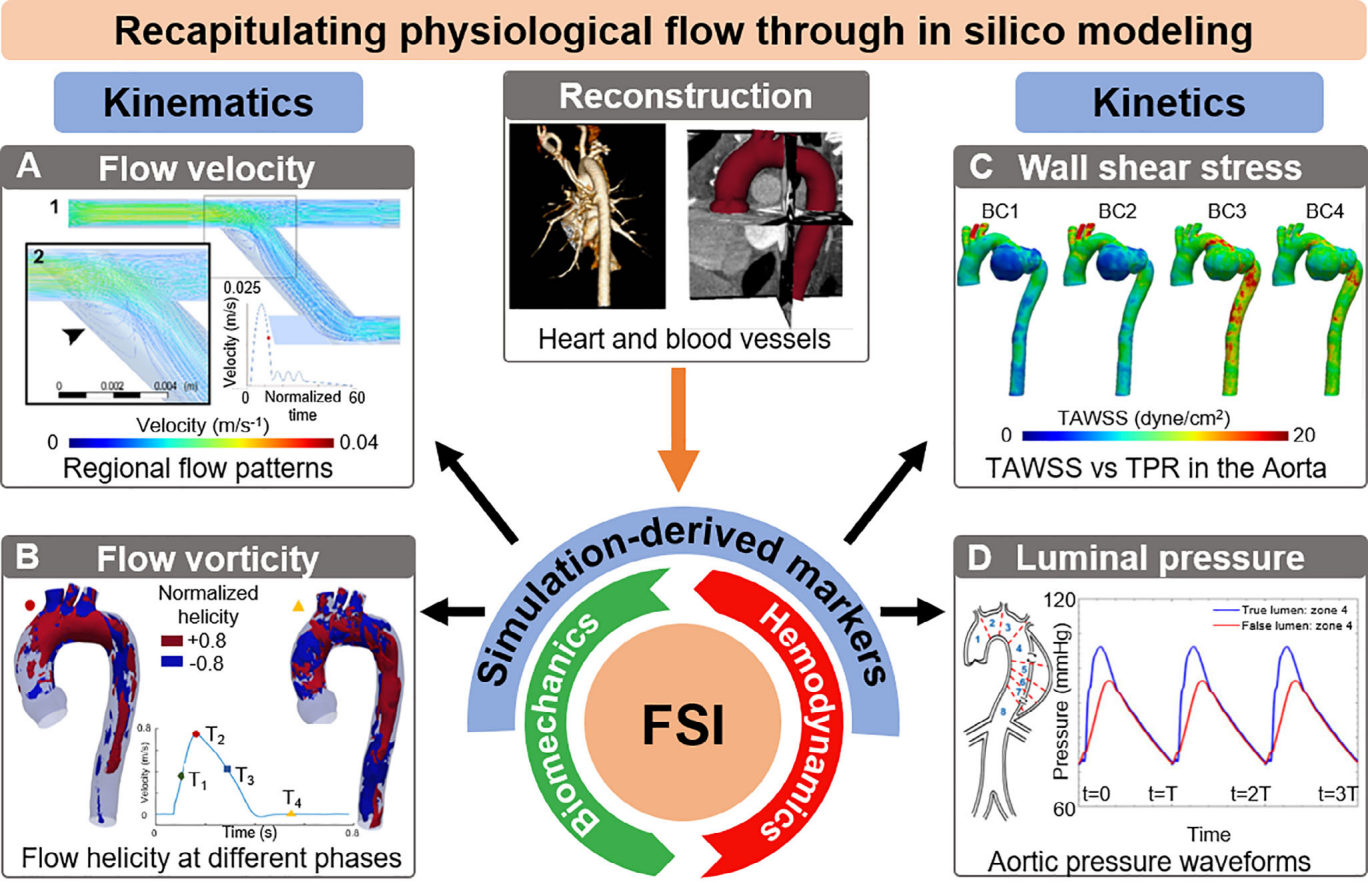
In silico fluid‐structure interactions (FSI) simulations of physiological flow used to establish potential markers of CV performance from subject‐specific geometric reconstruction. (A) Intraventricular flow velocity patterns acquired at a fixed timepoint and averaged over a phase of the cardiac cycle. (B) Aortic flow helicity at different phases of the cardiac cycle. (C) Time‐averaged wall shear stress (TAWSS) as a function of changing peripheral vessel resistance (TPR). (D) In silico recapitulation of in vitro aortic pressure waveforms. Reproduced with permission [43]. Copyright 2022, Frontiers. Reproduced with permission. [189] Copyright 2018, John Wiley and Sons. Reproduced with permission [190]. Copyright 2021, Institute of Physics.

Broadly speaking, computational modeling techniques often focus on recapitulating structural mechanics [159, 160, 161], fluid dynamics [162], and a combination of both [163]. While a wide range of experimental and computational modeling techniques have been applied to CV hemodynamics, each group, individually, faces several challenges. Experimental techniques are limited in their ability to replicate the complex structures and interactions present in native tissues, whereas in vivo computational models face challenges in predicting outcomes under parameter perturbations. Further, computational techniques require inputs from clinical data or experimental systems to build a faithful model of flow and to incorporate optimized parameters tailored to personalized conditions (Figure 3A) [164]. Most computational techniques are integrated with data derived from experimental methods, such as various imaging techniques, to improve modeling power (Figure 3B) [51, 59, 164, 165, 166]. The use of computational tools offers a unique opportunity to evaluate and optimize tissue engineering parameters for 3D bioprinting prior to in vitro studies. For instance, bioprinting process parameters have been tuned and optimized by computational methods to achieve adequate manufacturing fit and tolerance [167]. Computational tools can also assist in designing vessel geometries and determining flow parameters, mass transport, and biological function [49, 167, 168, 169]. Furthermore, the required mechanical properties of bioprinted constructs could be determined using computational simulations, enabling the creation of functional, mechanically robust tissues that can be customized to match target tissues and meet the specific needs of individual patients [170, 171]. This is particularly important when 3D bioprinting CV constructs from a target 3D computer‐aided design (CAD) model, in which the same CAD model can be fed into computational tools to run analyses in parallel with the in vitro experiments (Figure 4A).

### 4.2 Quantifying Blood Vessel Resistance to Replicate CV Hemodynamics

Finite element (FE) modeling of the cardiac muscle has provided substantial insight into the complexity of arterial and myocardial biomechanics [175, 176]. Various constitutive models exist, ranging from phenomenological transversely isotropic and neo‐Hookean models to more complex structure‐based models, such as the Ogden–Holzapfel model of the heart [176]. While the accuracy of each model in representing in vivo motion is open to debate, it remains unquestionable that integrating biomechanical models with CV flow is computationally intensive. Perhaps the least computationally expensive technique is lumped‐parameter modeling, in which the heart and vasculature are reduced to 0D compartmental models with no geometric parameters, and each compartment is modeled as a distinct functional approximation of whole‐body circulation, thus allowing an inexpensive assessment of CV physiology. For instance, the biomechanical heart model can be mathematically modeled as an ordinary differential equation (ODE), such as the Bestel–Clement–Sorine models [177]. More recently, 3D models of the heart and large arteries have been used alongside 0D compartmental models of the vessel tree. The distributed lumped‐parameter framework was introduced to model fluid‐structure interactions (FSI) using 1D equations. Using this approach, the authors reduced the computational cost of their FSI framework, with flow and pressure readings in agreement with the 3D benchmark [178, 179]. The 3D model of a biomechanical CV flow simulation consists of (i) a geometry, (ii) boundary conditions (BCs), and (iii) the governing equation [180]. In CV simulations, BCs are often modeled using electrical circuit analogies. While circuit resistance is analogous to the pressure drop across the vessel wall, capacitance is analogous to vessel elasticity or distensibility [181, 182]. For instance, the Windkessel effect in the large arteries can be interpreted as a RCR circuit [162, 178, 181]. Geometries are either assumed to be idealized versions or to be intricate, subject‐specific entities derived from medical imaging (discussed in Section 5).

### 4.3 Estimating Wall Shear Stress as a Coupled Metric of CV Biomechanics and Hemodynamics

A distinct phenomenon in CV flow is the coupled dynamics of the mechanical responses of the vessel tissue to blood flow loading, which define the in vivo biomechanical behavior of the vessel. Accordingly, a broad area of subject‐specific research focuses on investigating the interrelationship between blood flow and vessel function at the blood–tissue interface (Figure 4B,C). The functional relevance of these FSIs can be quantified through markers such as wall shear stress (WSS), vortex formation, fractional flow reserve, and other hemodynamic forces [163, 183, 184]. For instance, elevated WSS has been associated with the adaptive remodeling of compliant vessels, such as the coronary artery and the aorta, in atherosclerosis and aortic dissection, respectively. Various imaging‐based strategies have been proposed to delineate these parameters, with the characterization of mechanisms contributing to impaired biomechanical behavior of the vessels as a primary motivator. Advancements in medical imaging techniques have facilitated subject‐specific analyses through 3D geometry reconstruction. Subject‐specific geometries are coupled with FSI algorithms, such as the arbitrary Lagrange–Eulerian [163], immersed boundary [185, 186], and coupled‐FSI methods. While subject‐specific modeling has traditionally focused on major arteries, a growing body of research is evaluating the role of mechanical and hemodynamic forces within the CV microenvironment. Circuit‐based boundary conditions at vessel truncations are used to mimic downstream arteriolar resistance and compliance [187, 188].

These parameters are estimated from flow‐rate quantification via imaging and/or from pressure measurements during catheterization. In addition to using imaging data to quantify boundary conditions in FSI simulations, imaging techniques such as ultrasound elastography and phase‐contrast (PC) MRI are used to reduce assumptions in material properties by “directly” estimating the underlying material stiffness. For instance, Romarowski et al. developed a framework to reduce uncertainty in assumed material properties when simulating aortic FSI using PC‐MRI‐derived BCs [189]. In addition to using CT‐derived geometries of the aorta, the authors enforced the BCs using velocity profiles obtained from PC‐MRI. BCs were enforced by minimizing the error between CFD‐derived physical quantities and PC‐MRI data (Figure 4C). The authors addressed noise in PC‐MRI‐derived velocity data by applying a filter. By reinforcing the CFD model with PC‐MRI, the authors characterized the inlet and outflow velocity with high fidelity. Recently, Capellini et al. [190] and Calo et al. [191] suggested a moving‐boundary method that directly prescribes the aorta's deformation. The deformation of the aortic wall at each time step is quantified using a radial basis function (RBF)‐mesh morphing approach. Herein, the Euclidean distance between points in a source and a target region is minimized to obtain a unique solution for 3D displacements. Rather than assuming aorta‐specific material properties, the authors leverage information from CT images to enhance the fidelity of a general CFD formulation as an alternative to FSI. However, such methods are subject to limitations related to image quality, with low spatiotemporal resolution often resulting in noise and producing nonphysical displacements. Despite the limitations of image‐derived CFD and FSI models in accurately describing arterial flow, most studies involve multiscale phenomena that contribute to system‐level flow. The inclusion of both circuit‐based and imaging‐derived boundary conditions shows promise for characterizing flow patterns, with flow irregularities potentially serving as reproducible markers of CV etiologies (Figure 4D).

### 4.4 Identifying Disturbed Flow Patterns

Microfluidic investigations of flow parameters such as WSS, FSS, and oscillatory flow have widely reported that these factors influence cell growth and remodeling. Recent studies have reported the presence of secondary flow patterns, including laminar vortices, as contributors to endothelial dysfunction in 2D platforms of blood vessel systems [68]. Indeed, disturbances such as vortices within laminar flow systems, including most microfluidic patterns, may create low‐shear environments, thereby impeding cell growth and differentiation. Vortices have been confirmed in in vitro platforms by adding an array of ridges under peristaltic flow conditions. Computational studies show a direct correlation between ridge spacing and angle with the development of vortices. Recent explorations have enabled control of vortex strength and location both spatially and temporally, with such disturbances influencing the architecture of ECs and CMs. By maintaining low *Re*, laminar vortices have been observed to realign actin fibers within the cytoskeleton and effectuate changes in nucleus shape and structure within ECs [68]. The usage of ridge‐like arrays in generating disturbed flow patterns is gaining in popularity, thus distinguishing the effects of laminar and ’disturbed’ flow patterns on CV microfluidic systems. Morphological analysis has further revealed that regions of disturbed flow—such as recirculating vortices and flow reattachment zones—promote redistribution and often destabilization of EC adherens junctions, as evidenced by the intermittency or delocalization of VE‐cadherin at cell–cell contacts [192]. For example, sustained vortical shear or oscillatory wall‐shear stress leads to loss of continuous VE‐cadherin circumferential staining, increased permeability, and inflammatory activation [193]. In contrast, unidirectional laminar shear supports the stable linear arrangement of VE‐cadherin at junctions, maintaining strong cell–cell adhesion and barrier integrity [194]. These findings suggest that the hemodynamic microenvironment links flow pattern (laminar vs. disturbed), EC morphology (elongated and aligned vs. cobblestone and randomly oriented), and intercellular adhesion strength through modulation of junctional complexes and associated mechanosensitive signaling pathways such as KLF2, eNOS, and NF‐κB [192, 193].

## 5 Enhancement of In Vitro Modeling through Computational Simulations

**FIGURE 5:**
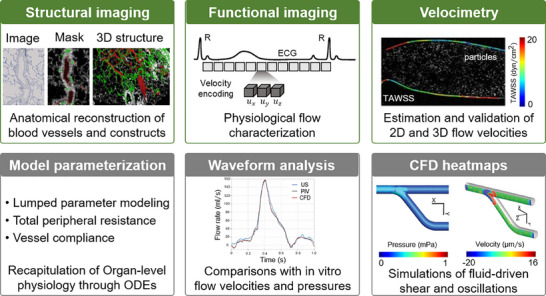
Application of imaging techniques in the development of computational flow models. Top: structural and functional imaging modalities provide ground‐truth data for anatomical and physiological function. While computed tomography (CT) captures vascular structure, velocimetry methods estimate flow‐related parameters. Bottom: in scenarios lacking catheterization data, reduced‐order models can approximate physiological pressures. CFD outputs, such as time‐averaged wall shear stress (TAWSS) and the oscillatory shear index, can complement traditional experimental markers, such as flow velocities, to enhance physiological insight. (top) Reproduced with permission [217]. Copyright 2019, Nature. Reproduced with permission [125], and (bottom) Reproduced with permossion [226]. Copyright 2022, Elsevier. Reproduced with permission [47]. Copyright 2024, John Wiley and Sons.

The utility of computational modeling techniques spans from understanding anatomical structures to pathological studies and the development and optimization of surgical plans. For example, in a study by Yevtushenko et al., CFD was used to train a neural network to predict hemodynamic parameters in patients with aortic stenosis [195]. Furthermore, the application of CFD in clinical interventions has been demonstrated by creating models of CHDs and evaluating the outcomes of different surgical approaches using these platforms [196]. In addition to surgical planning, computational models have been used to assess the impact of stent placement in CHDs, leading to the design and development of patient‐specific stents for optimal outcomes [197]. Such applications demonstrate the significance of in silico modeling techniques in understanding flow parameters in CV systems. While traditional models have been implemented at a macroscopic scale, a growing body of research is leveraging computational modeling to (i) guide the fabrication of bioprinted constructs, (ii) predict mechanical forces and investigate the corresponding cellular response, and (iii) predict cell viability [198, 199, 200] (Figure 5). The innovation of applying computational models to facilitate these applications largely stems from the adaptability and pliability of bioinks, which enable precise tissue fabrication. Structured bioinks, in particular, facilitate key CV properties such as electrical conduction and mechanotransduction, which are essential for functional tissue engineering. Computational analysis has been used as a predictive tool to identify factors contributing to cell viability [198, 199]. As described in previous sections, a wide range of in vitro, in vivo, and in silico models have also been developed to effectively determine flow characteristics in CV systems.

### 5.1 Leveraging Medical Imaging to Inform Computational Modeling of In Vitro Constructs

Computational models can provide insights into the flow dynamics of both healthy and diseased tissue and offer information on temporal alterations or responses to interventions. However, such models often require input from in vitro or in vivo observations to be accurate. The integration of experimental studies (including imaging and bioprinting) with computational models is crucial for accurately assessing flow dynamics in health and disease. Indeed, computational models are often combined with techniques such as catheterization, structural and functional imaging methods, to facilitate accurate measurements of model parameters [201, 202, 203]. 1D models have been developed by integrating the Navier–Stokes (NS) equations across the cross‐section of vessels. These models often represent blood vessels as a series of interconnected cylindrical structures. Being able to model wave propagation in blood vessels induced by interactions of the fluid with vessel walls, these models are useful in understanding the effect of geometrical alterations that could be caused by stenosis or the presence of stents and prosthetics in the aorta or other systemic arteries, or for studying the impact of vessel stiffness, causing complications [204, 205]. Furthermore, these models could be integrated with 0D models to model the arterial tree, including the biological construct. Generally, 0D models do not account for spatial variation and assume uniformity in pressure, flow, and volume across dimensions at specific locations and times. Such models represent the system as a series of electrical circuit analogs, with the parameters being the flow rate and the pressure difference. The parameters for 1D models can be obtained through in vivo catheterization or using ultrasound techniques in conjunction with PIV [206, 207].

While 2D models can also be applied to vasculature, accounting for radial variations in flow velocity, 3D models provide the highest degree of accuracy. Such models are based on imaging data obtained from experimental techniques such as MRI or CT scans and require data segmentation, followed by numerical calculations that treat blood vessels as elastically deformable structures. These models can simulate detailed flow patterns, pressure gradients, and WSS. MRI is a noninvasive medical imaging technique used to visualize internal structures with high resolution. The basic principle of MRI involves aligning the spins of protons with a powerful external magnetic field, perturbing their alignment with radiofrequency pulses, and measuring the relaxation time it takes for the protons to return to their initial state. This technique allows for detailed visualization of soft tissue structures, with a high degree of detail [208, 209]. Furthermore, the development of techniques such as 4D MRI enables the capture of the structure in 3D and its alterations over time, hence allowing the modeling of blood flow velocity across different measurement sections and directions within a single measurement, as well as nonlaminar flow profiles [210]. MRI has been widely used to analyze CV hemodynamics (Figure 5). In addition to the benefits of geometric reconstruction and design parameterization, MRI can be used to quantify in vivo flow characteristics. For example, Nakaji et al. demonstrated the use of MRI to study flow in the biventricular system [211]. Using a 4D MRI setup, normal flow patterns in the biventricular system of young, healthy individuals were analyzed, elucidating the natural physiology of these structures, and the feasibility of applying 4D MRI for detecting flow patterns and energy dynamics was demonstrated [211]. Similarly, Harloff et al. [212] used 4D MRI to determine blood flow velocities at the left and right carotid bifurcations in healthy individuals and patients with complications. In addition to its utility for determining flow characteristics, MRI has been integrated with computational methods to develop robust models of CV flow [212]. Saglietto et al. reported the use of high‐resolution MRI to obtain geometric models of lenticulostriate arteries and to develop a computational model of arterial fibrillation [213]. Similarly, MRI was used to generate CFD models, and together they were employed by Sundareswaran et al. for surgical planning of Fontan repair in patients with single‐ventricle congenital heart defects. This allowed for effective planning of the surgery concerning its hemodynamic outcome by evaluating hemodynamic parameters pre‐ and postsurgery [196]. The effectiveness of MRI‐based CFD for diagnosis and treatment planning has also been demonstrated in a separate study by Goubergrits et al., which showed that peak systolic pressure drops can be reliably determined using MRI‐based CFD in patients with CHDs [214]. However, 3D models require significant computational power, potentially limiting their direct application in clinical settings [215, 216]. In these modeling techniques, 0D models can simulate overall pressure and flow rate in a circulation system and serve as applicable boundary conditions for 3D models. 1D models, on the other hand, can improve the boundary conditions and simulate the pulse wave transmission.

#### 5.1.1 Image‐Based Methods Offer Subject Specificity

Many ultrasound techniques have been used to validate various computational models and to determine boundary conditions for CFD simulations that mimic patient‐specific in vitro conditions. Specifically, vascular ultrasound, similar to Doppler echocardiography, has been used for decades to assess cardiac properties such as velocity, directionality, and flow volume. Furthermore, by leveraging the Doppler effect, echocardiography can be used to determine myocardial tissue velocity (Figure 5). Integrating flow imaging techniques with computational models can provide a deeper understanding of flow dynamics across the entire domain. However, all these techniques rely on the experimental methods described in the previous sections to determine modeling parameters and validate the model. For example, CFD analysis of hemodynamics in mitral regurgitation has been evaluated against an in vitro‐developed pulsatile flow loop coupled with imaging chambers, validating the model's conclusions regarding pressure, velocity, and flow events and confirming CFD as a tool for analyzing mitral regurgitation [218]. Similarly, to address the variability observed among CFD models of internal carotid artery sidewall aneurysm (ICASA) developed by different groups, Yi et al. used PIV measurements to verify the flow characteristics predicted by the CFD models, including flow rate, vortex location, and flow velocity [219]. In addition, these computational methods have been integrated with in vitro experiments to analyze CV hemodynamics, validate the findings, and inform the optimization of in vitro constructs. This dramatically enhances the ability to replicate the inherently complex natural conditions in vitro. For example, the formation of arterial thrombosis is significantly affected by vessel geometry and blood flow conditions. Costa et al. reported the development of a microfluidic chip fabricated via stereolithography, based on CT angiography data, that replicates both healthy and stenotic arterial geometries. CFD analysis was used to evaluate shear rates associated with thrombosis, confirming the in vitro observations of the stenotic models [220]. Furthermore, when combined with in vitro analysis, computational modeling techniques have been used to evaluate intervention outcomes, optimize procedures, and design implants, such as stents. For example, to assess the effect of hemodynamics on the failure of large artery bypass grafts, Ku et al. developed an in vitro artery phantom with stenosis and bypass, and compared the outcomes of CFD and MRI analysis of flow characteristics [221]. Similarly, Li et al. assessed the applicability of computational modeling to stent deployment for treating intracranial aneurysms through integration with in vitro modeling techniques. This was performed by placing a silk flow‐diverting stent in a silicone aneurysm phantom, followed by PIV analysis and CFD modeling. It was demonstrated that, without the stent, the CFD model yielded more accurate results than PIV, indicating potential for further optimization of the modeling technique [222]. Comparison with catheterization techniques confirmed the reliability of the acquired data, demonstrating a noninvasive alternative for use in clinical settings [214].

#### 5.1.2 Flow Velocimetry Provides Rigorous Validation of Computational Models

Computational models of CV flow dynamics are susceptible to inconsistencies in reproducing flow parameters across studies and thus require rigorous validation protocols. Both Doppler echocardiography and flow MRI have been used to benchmark in vivo flow patterns and to assess the fidelity of computational models. For instance, Quaini et al. observed a discrepancy between the 3D isovelocity area computed by CFD and the 3D color Doppler in an in vitro study. The discrepancy was due to the strong dependence of the 3D color Doppler measurement technique on the Doppler angle [218]. Swanson et al. have also observed a discrepancy between velocity data measured by Doppler echocardiography and CFD in patients with CHD pre‐ and postrepair [165]. To overcome the shortcomings of conventional Doppler ultrasound, speckle tracking has been applied to vector flow imaging (VFI) [223]. The speckle tracking method uses a pattern‐matching algorithm to track the backscattered echoes produced when sound waves are scattered by blood and tissue, which remain relatively constant as blood cells and tissues move. Haniel et al. used ultrasound VFI to measure the monophasic recirculatory flow patterns in an in vitro study, which agreed with results computed from FSI [224]. Similarly, Jensen et al. observed flow patterns and vortices in VFI and CFD simulations of a healthy carotid bifurcation [225]. Another study by Hvid et al. reported overall agreement between VFI's measurement of the in‐plane fluid velocity profile and CFD results for intracardiac blood flow patterns [174]. However, despite advances in speckle tracking in ultrasound measurement, VFI has been shown to fall short of accurately measuring flow‐velocity magnitude in an in vitro study of an idealized quasi‐2D phantom of the human LV outflow tract [226].

Laser Doppler velocimetry (LDV) has emerged as a validation approach for benchmarking flow parameters in in vitro settings, surpassing standard PIV techniques. LDV relies on the frequency shift between the received and transmitted signal to quantify blood flow velocity. The reflected signal interferes with the transmitted signal, thereby altering the photocurrent in the photodetector. In general, laser Doppler velocimetry has higher resolution than ultrasound techniques, enabling investigations of microcirculation. A potential drawback is the long acquisition time required to obtain sufficient statistics for calculating the correct velocity profile [227]. Irregular flow within a restricted volume in vivo can also cause gradient broadening, thereby interfering with the Doppler shift, necessitating MRI techniques to improve measurement accuracy [228]. LDV has been used to validate various hemodynamic parameters computed using CFD in an in vitro model of an end‐to‐side vascular graft, such as flow velocity and WSS. Bauer et al. observed overall agreement between CFD and experimental results, with discrepancies when flow conditions were destabilizing, attributable to shortcomings in the numerical method [229]. Because of its accuracy in measuring absolute velocity components, LDV has also been used to validate complex patient‐specific numerical studies based on MRI‐derived data. For instance, Bauer et al. [229] and Castagna et al. [230] utilized LDV to measure WSS in settings of aneurysms and arteriosclerosis using a simple model of a hydraulic circuit (Figure 5). The results were validated in parallel with numerical results from CFD studies based on geometries derived from MRI imaging, which indicated good agreement between the LDV experimental technique and the numerical method. In addition to studying hemodynamic systems in fluidic circuits, CFD and LDV have been used to characterize the biological responses of CV cells in a bioreactor system under steady and time‐dependent WSS [48]. A cone‐and‐plate bioreactor system exposed cultured cells to a uniform shear stress, and a point‐to‐point comparison of tangential velocity measured by LDV and computed using CFD showed excellent agreement.

### 5.2 Emerging Techniques

#### 5.2.1 Advancing Fabrication through Computationally‐Guided Tissue Engineering

**FIGURE 6:**
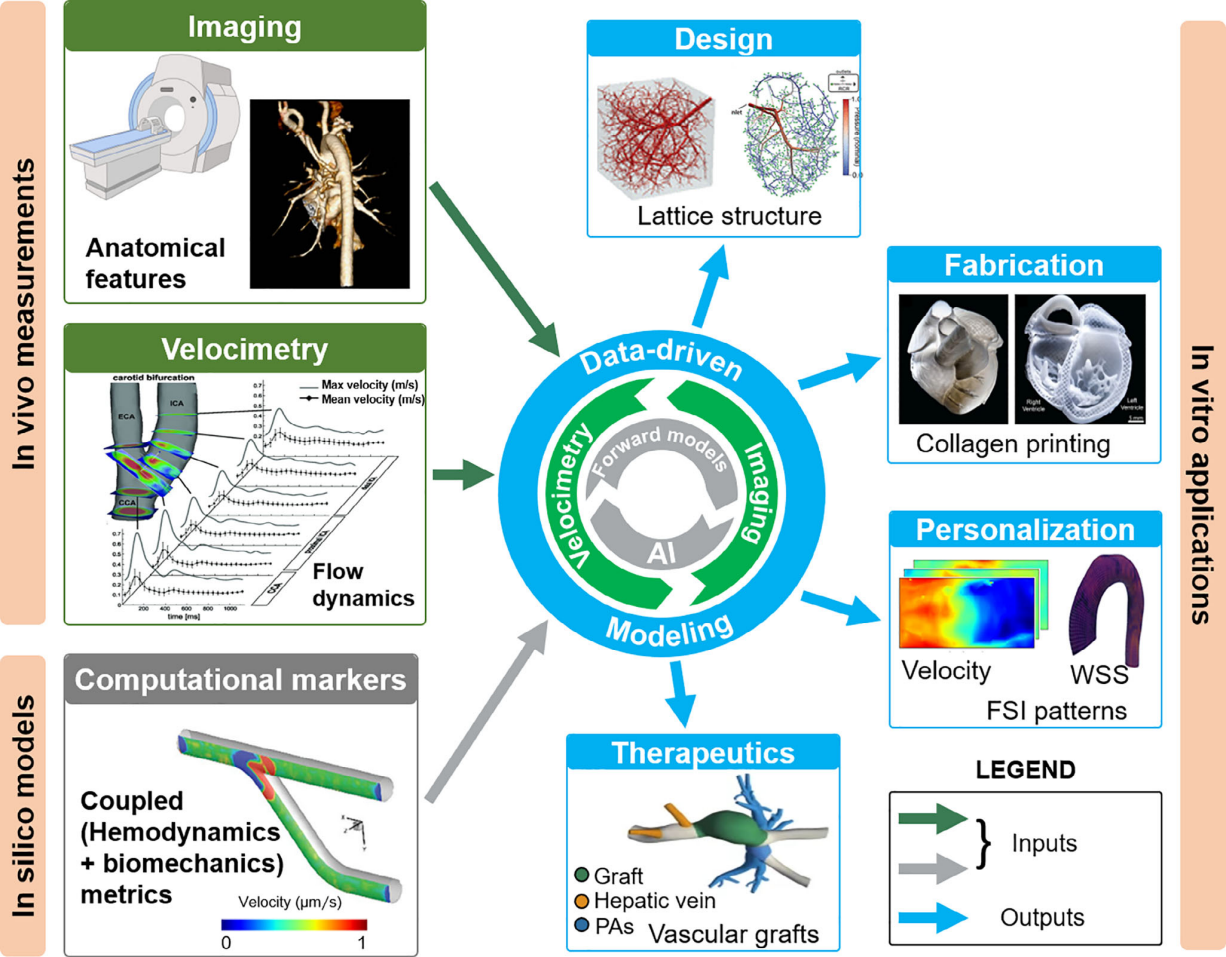
Schematic illustrating the emerging trends in the integration of imaging and computational parameters as inputs for data‐driven modeling. These models can be leveraged across various applications to enhance the design and optimization of biological constructs for CV flow. Key applications including design, fabrication, model personalization, and therapeutics are highlighted. PA: pulmonary artery, WSS: wall shear stress, AI: artificial intelligence.Reproduced with permission [43]. Copyright 2022, Frontiers. Reproduced with permission [234]. Copyright 2025, Science. Reproduced with permission [233]. Copyright 2019, Science. Reproduced with permission [239]. Copyright 2022, Frontier. Reproduced with permission [236]. Copyright 2021, Springer Nature.

Beyond scaffolds, more advanced tissue engineering techniques, such as bioprinting, have recently been used in combination with computational strategies to develop more accurate hemodynamic models [30]. This can be considered an essential step, as such strategies can closely mimic the patient‐specific 3D physiological environment. However, their use remains limited, underscoring a clear need for further research on advanced models [36]. Recent advancements in integrated experimental–computational modeling studies have emerged as a means to guide applications beyond understanding and recapitulating disease mechanisms (Figure 6). Importantly, bioink fabrication can be scaled up through a growing body of research on computational intelligence for manufacturing. In particular, the development of computational models that elucidate structural anisotropy, mechanical stresses, and shear moduli has been identified as a feasible pathway for advancing biofabrication. For instance, Egan and co‐workers introduced a computational protocol to design lattice structures and simulate mechanical properties of 3D scaffolds for orthopedic applications [231]. This involved topology optimization to create cubes and octets and to simultaneously tune their strength and shear moduli, thereby facilitating bone growth. Computational simulations were used to optimize the lattices that comprised the scaffolds, ultimately aiming to preserve the buckling of the printed structures in the context of spinal fusion. Along the same lines, Jia et al. customized the architecture of 3D bioprinted constructs by optimizing the distribution of mechanical stresses within the constructs and validated the approach using experimental models. By simulating various growth and remodeling scenarios, the authors highlighted the benefits of computational parameterization in recapitulating the biological function of tissue [232]. The use of computational intelligence in supporting biofabrication has since extended to CV applications. Lee et al. introduced a technique for building complex collagen scaffolds to develop bioprinted heart models using human iPSCs across various length scales [233]. They integrated MRI, which provides tissue‐level anatomical data (e.g., trabeculae, valves), with a computational space‐filling algorithm to create a dense network of blood vessels. The underlying lattice structure informed the creation of a bioprinted human heart model that could potentially recapitulate mechanical and biological properties. Most recently, Sexton et al. addressed the inherent complexities of computationally determining physiologically relevant vessel networks using CFD analysis [234]. Their applications focused on generating rapid vessels to facilitate large‐scale production of bioprinted CV models. Their study combined standard hemodynamic transport parameterization with lattice design to generate anatomically faithful bioprinted heart models, highlighting key synergies between computational and experimental in vitro modeling.

In addition to advancing the physiological relevance of bioprinting, the integration of computational and experimental methods has been utilized to inform therapeutic development. Specifically, hemodynamic modeling has also been used in regenerative strategies to personalize the development of tissue‐engineered vascular grafts [235]. Schwarz et al. developed patient‐specific CFD models based on MRI images of Fontan patients [236]. Following this, the tissue‐engineered graft scaffolds composed of polyglycolic acid and polycaprolactone/lactic acid were tuned for stiffness, and the graft performance was analyzed in silico, demonstrating the applicability of CFD to the design and development of tissue‐engineered constructs. Similarly, Yeung et al. used patient‐specific MRI data to 3D print branched pulmonary artery grafts [237]. The hemodynamic parameters analyzed included power loss, flow distribution, and WSS, and the CFD‐optimized design replicated physiological patient‐specific hemodynamics [237]. While further studies are required on the regions susceptible to thrombosis, this study demonstrated the applicability of computational modeling techniques to improve the design and engineering of vascular grafts. The combination of computational methods and tissue engineering strategies has also extended to the development of models for disease prediction. This is demonstrated by Lee et al., who developed an endothelialized tissue‐engineered model of early atherosclerosis in vitro and combined it with PIV and CFD modeling to evaluate disease progression and possible interventions, demonstrating the potential impact of integrating computational and experimental methods in preclinical evaluations [238]. In essence, experimental–computational simulations for in vitro CV models can be leveraged to guide biofabrication and promote personalization through the elevating properties associated with (i) structural integrity, (ii) flow transport, and (iii) biocompatibility of the tissue‐engineered constructs.

#### 5.2.2 Artificial Intelligence for Predictive Subject‐Specific Disease Model Development

The scope of artificial intelligence in healthcare is rapidly growing, with applications in clinical diagnosis deemed to be limitless. While machine learning (ML) techniques such as convolutional neural networks (CNN), specifically U‐Net, are used to automate image segmentation and reduce human error, a growing body of research has applied ML to enhance quantitative investigations of flow characteristics at multiple scales in CV biology [183, 240, 241]. Studies apply key ML algorithms to improve the predictive power of computational models in estimating flow parameters. A primary motivation for ML‐based approaches is to reduce the computational burden associated with high‐fidelity CV simulations by serving as surrogate models [242]. By learning relationships between anatomy, BCs, and flow‐related quantities, these methods can enable more rapid estimation of hemodynamic metrics, improving clinical integration. Liang et al. conducted a feasibility study to evaluate the accuracy of deep neural networks (DNNs) in reproducing the steady‐state hemodynamics of the thoracic aorta [243]. An encoder–decoder platform was implemented, featuring a shape encoder to represent the geometric coordinates of a subject‐specific aorta and a field encoder to represent scalars such as pressure and velocity. Despite the promise of DNNs to closely recapitulate flow patterns observed in conventional CFD analyses, the authors note several simplifications in the study. For instance, only flow and pressure readings were used for training and validation (e.g., WSS and OSI were not used). However, the performance of any DNN is often exacerbated by high‐dimensional input and output features. The application of more sophisticated layers may enable the integration of FSI features, such as WSS, thereby contributing to a more advanced diagnostic tool. Ferdian et al. applied a similar approach, differing from the previous study through the additional encoding of a WSS layer, termed WSSNet [239]. Despite its promise to generalize WSS patterns across the test set, the study suffers from similar limitations, in which the geometries and BCs are restricted to rigid walls and constant flow inputs, respectively. Another emerging tool for leveraging ML in the computational modeling of CV flow is the physics‐informed neural network (PINN) [244]. PINNs can be trained using sparse datasets, with physical laws enforcing relationships across the domain rather than relying on explicit neighborhood information. This reduces dependence on data density and measurement quality. Furthermore, CV flow phenomena can be captured using physics‐informed loss functions, thereby reducing the need for explicitly prescribed inlet or outlet flow or pressure profiles in the training set. Arzani et al. implemented PINNs to quantify WSS in idealized geometries of large arteries [245]. The loss function is defined using the problem‐specific implementations of the 3D NS equations and the corresponding BCs. Thus, the loss function can be defined as the linear combination of BCs, governing equations, and experimental validation to obtain a unique solution. The resulting WSS showed excellent agreement with CFD simulations, indicating that the study holds immense promise for extending the model to subject‐specific geometries. The use of PINNs and other physics‐informed computational models is particularly useful in settings with limited access to clinical data. This could be especially true in CHD settings, where imaging and electrophysiological data are rarely reproducible [246]. Despite their potential advantages, training PINNs remains computationally demanding and, in certain instances, can be as computationally intensive as or more so than conventional FSI analyses [247].

Ideally, any ANN can be trained by leveraging the fidelity of CNNs for encoding spatial features, combined with that of PINNs for encoding functional features, to obtain a robust, generalized solution for CV flow in health and disease. Recent models leverage deep generative learning to address limited data availability. In essence, these models provide a scientific/statistical representation of the distribution of scarce data (e.g., medical images, velocimetry) by creating a vast repository of forward simulations without the need for intricate physics‐based modeling. For example, generative models such as generative adversarial networks and variational autoencoders can be trained on a few FSI models of a bioprinted vessel network and simultaneously generate hundreds of unique flow parameters (e.g., WSS, vortices) that vary in mechanical and biological properties, thereby denoting the vessel structure. As such, a generalized model of the bioprinted vessel can be applied to existing CHDs and develop a scalable computational model for in vitro applications. Generative models have been tested in medical image reconstruction [248], risk stratification [249], and even in tumor modeling [250]. Thus, these predictions of key CV design, flow, and mechanical parameters can be tuned to improve the overall fidelity of 3D biological constructs in biofabrication, recapitulate physiological flow, predict cell–cell interactions and viability, and potentially generalize computational models across a variety of CVDs and CHDs.

## 6 Conclusion

In summary, the ability to model CV hemodynamics has significantly advanced CV research by enabling deeper mechanistic insight and improved predictive capability. Although experimental and computational approaches are often treated as distinct paradigms, this review highlights that their integration is essential for faithfully recapitulating CV physiology. Consequently, many studies have adopted hybrid strategies, ranging from coupling in vivo flow measurements with computational frameworks to using advanced imaging modalities such as MRI and CT for anatomically informed modeling, to integrating bioprinted in vitro constructs with velocimetry and numerical simulations. Together, these approaches have emerged as powerful tools for capturing CV structure–function relationships. Collectively, they have demonstrated enhanced accuracy in assessing pathological conditions and stratifying cardiovascular risk, underscoring the critical role of computational strategies in advancing in vitro modeling toward clinical relevance. Nevertheless, persistent challenges remain, particularly regarding intermodel variability and uncertainty in predicted outcomes. Addressing these limitations will require incorporating state‐of‐the‐art data‐driven methodologies, including artificial intelligence, to guide biofabrication, enable personalization, and inform therapeutic development. By generalizing computational models of in vitro platforms, a scalable pathway to translate patient‐specific CV data can be achieved, enabling predictive design across CVDs and CHDs. Ultimately, the convergence of experimental fidelity in bioprinting with computational rigor represents a pivotal step toward the next generation of CV modeling and positions in vitro platforms as feasible, predictive tools for clinical decision‐making.

## Conflicts of Interest

The authors declare no conflict of interest.
